# A Multivariate Model for Prediction of Obstructive Coronary Disease in Patients with Acute Chest Pain: Development and Validation

**DOI:** 10.5935/abc.20170037

**Published:** 2017-04

**Authors:** Luis Cláudio Lemos Correia, Maurício Cerqueira, Manuela Carvalhal, Felipe Ferreira, Guilherme Garcia, André Barcelos da Silva, Nicole de Sá, Fernanda Lopes, Ana Clara Barcelos, Márcia Noya-Rabelo

**Affiliations:** 1 Escola Bahiana de Medicina e Saúde Pública; Salvador, BA - Brazil; 2 Hospital São Rafael, Salvador, BA - Brazil

**Keywords:** CoronaryArtery Disease, Methods, Chest Pain, Models Statistical, Coronary Angiography, Troponin, Electrocardiography

## Abstract

**Background:**

Currently, there is no validated multivariate model to predict probability of obstructive coronary disease in patients with acute chest pain.

**Objective:**

To develop and validate a multivariate model to predict coronary artery disease (CAD) based on variables assessed at admission to the coronary care unit (CCU) due to acute chest pain.

**Methods:**

A total of 470 patients were studied, 370 utilized as the derivation sample and the subsequent 100 patients as the validation sample. As the reference standard, angiography was required to rule in CAD (stenosis ≥ 70%), while either angiography or a negative noninvasive test could be used to rule it out. As predictors, 13 baseline variables related to medical history, 14 characteristics of chest discomfort, and eight variables from physical examination or laboratory tests were tested.

**Results:**

The prevalence of CAD was 48%. By logistic regression, six variables remained independent predictors of CAD: age, male gender, relief with nitrate, signs of heart failure, positive electrocardiogram, and troponin. The area under the curve (AUC) of this final model was 0.80 (95% confidence interval [95%CI] = 0.75 - 0.84) in the derivation sample and 0.86 (95%CI = 0.79 - 0.93) in the validation sample. Hosmer-Lemeshow's test indicated good calibration in both samples (p = 0.98 and p = 0.23, respectively). Compared with a basic model containing electrocardiogram and troponin, the full model provided an AUC increment of 0.07 in both derivation (p = 0.0002) and validation (p = 0.039) samples. Integrated discrimination improvement was 0.09 in both derivation (p < 0.001) and validation (p < 0.0015) samples.

**Conclusion:**

A multivariate model was derived and validated as an accurate tool for estimating the pretest probability of CAD in patients with acute chest pain.

## Introduction

Acute chest pain is one of the most common reasons for emergency department visits. Since it may represent a clinical manifestation of cardiac ischemia, patient discharge is normally conditioned to a negative test for obstructive coronary artery disease (CAD).^1^ However, the efficiency of this defensive strategy is challenged by a low yield of cardiac tests, since only a portion of patients ends up having obstructive CAD and a smaller part will need revascularization.^2^ In addition, routine testing is not supported by evidence of beneficial effect^3^ and may have unintentional consequences: overdiagnosis and overtreatment of coronary disease not causally related to symptoms, prolonged hospital stay, unnecessary invasive procedures due to false-positive test results, and increased medical expenses.^4^

Therefore, a more rational approach is to indicate additional tests on the basis of pretest probability. Traditionally, this pretest evaluation is restricted to electrocardiogram and necrosis markers. However, the use of a multivariate model has the potential to improve accuracy and provide a more continuous range of probabilities. In order to develop and validate a multivariate model to predict CAD based on variables assessed at admission to the coronary care unit, 370 consecutive patients were studied. Thirty-five variables were tested as candidate predictors of obstructive CAD in order to generate a final model that was further validated in a subsequent sample of 100 patients.

## Methods

### Sample selection

During a period of 30 consecutive months, all patients admitted to the coronary care unit of our hospital were included in the study. Admission took place whenever medical judgment recognized any chance of a coronary etiology, regardless of electrocardiogram or troponin. The only exclusion criterion was the patient's decline to participate. As defined *a priori,* the first 370 patients were utilized as the derivation sample and the next 100 patients as the validation sample. The study was approved by an institutional review committee, and all the subjects gave informed consent to participate.

### Predictors of obstructive CAD

At baseline admission, three sets of variables were recorded as candidates for prediction of obstructive CAD. The first comprised 13 variables related to medical history, such as age, gender, previous history of CAD, risk factors for CAD, and comorbidities; the second set included 14 characteristics of chest discomfort; and the third set was composed of eight variables related to either physical examination or basic admission tests, including physical and radiologic signs of left heart failure, ischemic electrocardiographic changes (T wave inversion ≥ 1 mm or dynamic ST deviation ≥ 0.5 mm), positive troponin (> 99th percentile of the general population; Ortho-Clinical Diagnostics, Rochester, NY, USA), N-terminal pro-B-type natriuretic peptide (NT-proBNP, enzyme-linked fluorescent assay, Biomérieux, France), high-sensitivity C-reactive protein (CRP; nephelometry, Dade-Behring, USA), white cell count, plasma glucose, and hemoglobin. Laboratory tests were performed in plasma material collected at presentation to the emergency room. The medical history and chest pain characteristics were recorded by three investigators (M.C., A.M.C., and R.B.) trained to interview the patients in a systematic form, in order to decrease bias and improve reproducibility. Radiologic signs of ventricular failure and electrocardiogram were all interpreted by the same senior investigator (L.C.).

### Outcomes definition

The primary outcome to be predicted by the model was a diagnosis of obstructive CAD, defined by subsequent tests performed during hospital stay. The outcome data were collected by three investigators (M.C., A.M.C., and R.B.) and adjudicated by a fourth investigator (L.C.). For diagnostic evaluation, the patients underwent invasive coronary angiography or a provocative noninvasive test (perfusion magnetic resonance imaging, nuclear single-photon emission computed tomography or stress-echocardiography with dobutamine), at the discretion of the assistant cardiologist. In the case of a positive noninvasive test, the patients had angiography for confirmation. Based on this diagnostic algorithm, obstructive CAD was defined as a ≥ 70% stenosis on angiography. A normal noninvasive test (ischemic defect size < 5% of the left ventricular myocardium) indicated the absence of obstructive CAD and no further test was required. Regardless of coronary tests, the patients were classified as presenting no obstructive CAD if one of the following dominant diagnoses was confirmed by image: pericarditis, pulmonary embolism, aortic dissection or pneumonia. Secondarily, the model was tested for the prediction of death within 30 days of admission.

### Statistical analysis

**Figure 1:**
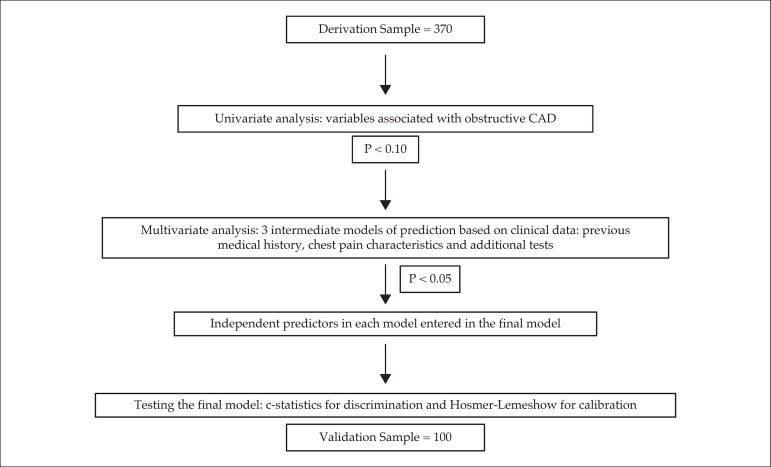
Flowchart of the statistical analysis.

The statistical analysis is depicted in Figure 1. The initial sample of 370 consecutive patients was utilized for the derivation of the model. First, univariate associations between obstructive CAD and baseline characteristics were tested by unpaired Student's *t* test for numeric variables and Pearson's chi-square test for categorical variables. Numeric variables not normally distributed were expressed as median and interquartile range and compared by nonparametric Mann-Whitney's test. Second, variables with a p value < 0.10 in the univariate analysis were included in the multivariate logistic regression analysis for prediction of obstructive CAD.

Multivariate models were developed by the stepwise method, forcing all selected variables into the regression and eliminating the least significant at each step, according to Wald's statistical test. Initially, three intermediate models were built, according to the type of predictive variables (medical history, chest pain characteristics or physical examination/laboratory tests). Independent predictors (p < 0.05) in each intermediate model were included as covariates in the final model. This final model was built hierarchically, with the order of variable imputation defined by clinical reasoning. The improvement of the model at each step was described by the decrease in -2Log likelihood.

Discrimination was evaluated by the area under the receiver operating characteristic (ROC) curve (AUC), while calibration was assessed by Hosmer-Lemeshow's test and correlation between predictive and observed prevalence of disease according to deciles of prediction. The incremental value of the full model in relation to the most basic model was evaluated by comparing the two AUCs by DeLong's test. In addition, *integrated discrimination improvement* by the full model was described according to Pencina's method.^5^

Subsequently, 100 consecutive patients served as the validation sample. In this sample, discrimination of CAD was tested by the AUC. Since calibration analysis by deciles would not be appropriate in a sample of 100 patients, observed CAD prevalence was compared among tertiles of CAD prediction. The incremental value of the full model in relation to the most basic model was evaluated by comparing the two AUC by DeLong's test. *Integrated discrimination improvement* by the full model was also described in this sample.

In a sensitivity analysis, the full sample of 470 patients was used to test whether the performance of the model changed according to the presence or absence of electrocardiographic or troponin changes. For this analysis, an interaction term was tested by logistic regression. The full sample was also used to test the prognostic value of the model. The AUC for 30-day mortality prediction was described and compared with the GRACE score^6^ as a proxy of a model specifically created for a prognostic purpose. DeLong's test was used to compare the AUCs.

Statistical significance was defined as alpha < 0.05. For numerical variables with normal distribution, mean and standard deviation was used, while a non-normal distribution implied in the use of median and interquartile range. SPSS, version 21.0, was the software used for statistical analysis.

### Acute chest pain score

In order to generate a score for CAD prediction, points were attributed to each positive variable, proportional to their regression coefficients in the final model. The prevalence of obstructive CAD was described according to score's deciles. Alternatively, the final regression formula was used to create a logistic calculator, provided as an Excel spreadsheet (electronic file) or application for smartphones (to be available in the near future).

### Sample size determination

As described above, two consecutive samples of patients were selected: the derivation set and the validation set. For the derivation set, the sample size was planned to allow inclusion of at least 10 covariates in the logistic regression model. The calculation was based on the following assumptions: 30% prevalence of obstructive CAD and the need for 10 events for each covariate in the logistic regression model.^7^ Therefore, a minimum of 300 patients would be required, and as a safety precaution, we planned to include a total of 370 individuals. The validation sample was set to test the discriminatory accuracy by the ROC curve analysis. Based on the assumption of an AUC of 0.70, to provide 90% power to reject the null hypothesis of an AUC equal 0.50, under the alpha of 5%, a minimum of 85 patients was required. Therefore, we planned to include 100 patients in the validation set.

## Results

### Sample population for model derivation

In total, 370 patients were studied, aged 60 ± 16 years, 57% males, 33% with a previous history of coronary disease. The median time elapsed between the onset of symptoms and first clinical evaluation in the hospital was 4 hours (interquartile range = 1.8 - 13 hours). At presentation, 52% of the patients had ischemic changes on the electrocardiogram, and 48% had positive troponin. Further investigation according to study protocol identified obstructive CAD in 176 patients, a prevalence of 48%. All cases had diagnostic confirmation by invasive coronary angiography. Regarding the 194 patients without CAD, 74 were classified by a negative angiography, 105 by a negative noninvasive test and 15 had another dominant diagnosis (four with pulmonary embolism, two with aortic dissection, seven with pericarditis, and two with pneumonia).

### Predictors of obstructive CAD

Among the 13 variables related to medical history, only four were associated with obstructive CAD: older age, higher prevalence of male gender, previous history of CAD, and a trend towards more diabetes (Table 1). When these four variables were included in the logistic regression, age and male gender remained statistically significant (Intermediate Model 1) (Table 2).

**Table 1:** Comparison of medical history, chest pain characteristics, and laboratory tests between patients with and without obstructive coronary artery disease

	Obstructive Coronary Disease		p Value
	Yes (n = 176)	No (n = 194)	
**Medical History**			
Age (years)	63 ± 14	57 ± 16	< 0.001
Male gender	121 (69%)	90 (46%)	< 0.001
Body mass index (kg/m2)	28 ± 4.8	28 ± 5.9	0.61
History of CAD	68 (39%)	55 (28%)	0.03
Diabetes	62 (36%)	51 (26%)	0.05
Hypertension	122 (70%)	138 (71%)	0.83
Current smoking	22 (13%)	18 (9.3%)	0.30
LDL cholesterol (mg/dL)	113 ± 64	116 ± 87	0.72
Family history of CAD	48 (28%)	42 (22%)	0.19
Chronic renal disease	9 (5.3%)	7 (3.6%)	0.45
Plasma creatinine (mg/dL)	0.95 (0.80 - 1.20)	0.80 (0.70 - 1.15)	0.10
Current statin therapy	85 (49%)	91 (47%)	0.71
Current aspirin therapy	75 (43%)	76 (39%)	0.44
**Chest Pain Characteristics**			
Left side location	137 (79%)	156 (81%)	0.70
Oppressive nature	97 (57%)	95 (49%)	0.14
Irradiation to neck	39 (23%)	51 (26%)	0.42
Irradiation to left arm	57 (33%)	53 (27%)	0.24
Vagal symptoms	61 (36%)	78 (40%)	0.35
Number of episodes	1 (1 - 2)	1 (1 - 3)	0.81
Duration (minutes)	40 (15 - 120)	40 (10 - 150)	0.82
Intensity (1 - 10 scale)	7.4 ± 2.5	7.1 ± 2.6	0.31
Relief with nitrate	84 (50%)	72 (37%)	0.02
Similar to previous infarction	70 (42%)	63 (33%)	0.08
Worsening with compression	7 (4.1%)	26 (13%)	0.002
Worsening with position	24 (14%)	36 (19%)	0.23
Worsening with arm movement	7 (4.0%)	16 (8.2%)	0.097
Worsening with deep breath	13 (7.5%)	36 (19%)	0.002
**Laboratory Tests at Admission**			
Ischemic changes on ECG	120 (68%)	73 (38%)	< 0.001
Positive troponin	116 (66%)	60 (31%)	< 0.001
X-ray and clinical signs of LVF	26 (15%)	5 (2.6%)	< 0.001
NT-proBNP (pg/mL)	363 (105 - 1850)	57 (20 - 235)	< 0.001
Plasma glucose (mg/dL)	120 (97 - 189)	112 (92 - 145)	0.22
C-reactive protein (mg/L)	7.3 (2.3 - 15)	5.7 (1.4 - 15)	0.09
White cell count	8.790 ± 4.300	7.701 ± 2.865	0.004
Hemoglobin (g/dL)	14.1 ± 1.9	13.7 ± 1.7	0.06

CAD: coronary artery disease; LVF: left ventricular failure. A family history of CAD implies in the presentation of the disease in a first-degree relative before the age of 55 years (females) or 45 years (males).

**Table 2:** Intermediates logistic regression models of medical history (Model 1), chest pain characteristics (Model 2) and laboratory tests (Model 3)

Variables	Multivariate significance level
**Model 1 (medical history)**	
Male gender	< 0.001
Age (years)	< 0.001
Diabetes	0.10
HDL cholesterol	0.35
Previous CAD	0.84
Plasma creatinine (mg/dL)	0.95
**Model 2 (pain characteristics)**	
Sensible to manual compression	0.024
Sensible to deep breath	0.037
Relief with nitrate	0.045
Similar to a previous MI	0.17
Sensible to arm movement	0.57
**Model 3 (laboratory tests)**	
Ischemic changes on ECG	< 0.001
Positive troponin	< 0.001
X-ray or clinical signs of LVF	0.016
White cell count	0.29
Hemoglobin (g/dL)	0.67
NT-proBNP (pg/mL)	0.81
C-reactive protein (mg/L)	0.70

MI: myocardial infarction; CAD: coronary artery disease; LVF: left ventricular failure.

Regarding chest pain characteristics, among 14 variables, only five had an association with CAD: relief with nitrates and similarity with previous myocardial infarction. On the other hand, worsening with manual compression, deep breath or arm movement were each more common in patients without CAD (Table 1). Of these, relief with nitrates, worsening with manual compression and with deep breath were the three independent predictors in the Intermediate Model 2 (Table 2).

Among the physical examination and laboratory tests, most variables were associated with CAD: ischemic electrocardiogram, positive troponin, and signs of left heart failure were more prevalent in patients with CAD. Also, four numeric variables had higher values in patients with CAD: NT-proBNP, CRP, white cell count, and hemoglobin (Table 1). In the Intermediate Model 3, ischemic electrocardiogram, positive troponin, and signs of left heart failure were the independent predictors (Table 2).

### Development of a model for CAD prediction

The eight variables independently associated with CAD in the Intermediate Models 1, 2, and 3 were candidates to the final model, which was built hierarchically in seven steps, defined by clinical reasoning: the first step comprised electrocardiogram and troponin together, followed by the second step that included left ventricular failure. These two first steps represented the severity of the clinical presentation. The third and fourth steps represented intrinsic characteristics of the patients, age, and gender. The fifth, sixth, and seventh steps were related to characteristics of chest pain, which were chosen to be last because of their subjectivity in clinical practice.

The first step of electrocardiogram and troponin had a -2Log likelihood of 437 (χ^2^ = 69, p < 0.001), which sequentially improved by the inclusion of left ventricular failure (-2Log likelihood = 427, χ^2^ = 9.8, p = 0.002), age (-2Log likelihood = 422, χ^2^ = 4.9, p = 0.02), gender (-2Log likelihood = 401, χ^2^ = 21, p < 0.001), and relief with nitrates (-2Log likelihood = 394, χ^2^ = 6.8, p = 0.009). The inclusion of worsening with manual compression (-2 log likelihood = 391, χ^2^ = 3.2, p = 0.07) and worsening with deep breath (-2 log likelihood = 389, χ^2^ = 2.3, p = 0.13) did not promote further improvement in the model. Therefore, the first six variables constituted the final model.

**Figure 2:**
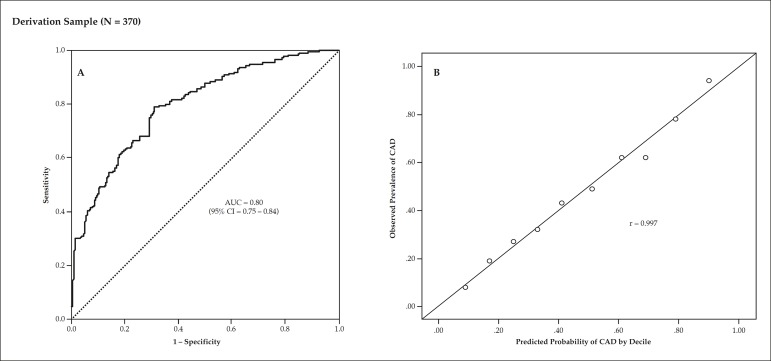
Analysis of the model's discrimination and calibration in the derivation sample of 370 patients. Panel A shows significant AUC of the probabilistic model for prediction of obstructive coronary artery disease. Panel B shows a significant correlation between predicted and observed probability of coronary artery disease (CAD). AUC denotes area under the receiver operating characteristic curve.

The final model presented good discrimination, with an AUC of 0.80 (95%CI = 0.75 - 0.84) (Figure 2A). The Hosmer-Lemeshow's χ^2^ of 1.95 indicated that the model was well calibrated (p = 0.98), as shown in the scatter plot of predictive probability *versus* observed prevalence of CAD by deciles (r = 0.99) (Figure 2B). The probability of CAD according to the final model ranged from a minimum of 3% to a maximum of 98%, with patients equally distributed across probabilities. Odds ratio, 95%CIs and regression coefficients, and p values of the final model are depicted in Table 3.

**Table 3:** Final model of logistic regression defining the independent predictors of obstructive coronary artery disease

Variables	Beta	Odds Ratio (95%CI)	p Value
Age (each year)	0.025	1.03 (1.01 - 1.04)	0.003
Relief with nitrates	0.60	1.8 (1.1 - 3.0)	0.016
Ischemic ECG	1.10	3.0 (1.9 - 4.9)	< 0.001
Positive troponin	1.15	3.2 (1.9 - 5.1)	< 0.001
Male gender	1.16	3.2 (1.9 - 5.3)	< 0.001
Signs of LVF	1.55	4.7 (1.6 - 14)	0.004
Sensible to deep breath	----	----	0.06
Sensible to manual compression	----	----	0.18

LVF: left ventricular failure.

### Incremental value of the full model

The AUC improved from 0.73 in the first model containing only electrocardiogram and troponin to 0.80 in the full model (95%CI of difference between the areas = 0.03 - 0.10, p = 0.0002). Discrimination progressively improved as variables were added: the AUC was 0.74 in the second model (adding left ventricular failure), 0.76 in the third model (adding age), and 0.79 in the fourth model (adding gender). The integrated discrimination improvement provided by the full model in relation to the first model was 0.09 (p < 0.001), a result of 0.05 of mean increase of probabilities in the group with events plus 0.04 of mean decrease of probabilities in the group free of events.

### Validation by the independent sample

**Figure 3:**
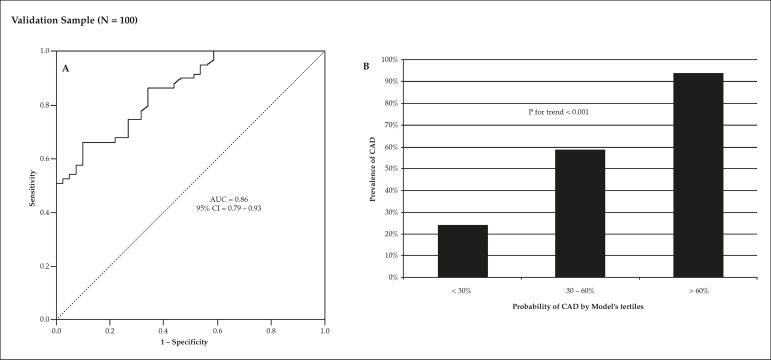
Analysis of the model's performance in the independent validation sample of 100 patients. Panel A shows a significant AUC of the probabilistic model for prediction of obstructive coronary artery disease (CAD). Panel B indicates a progressive increase in the prevalence of CAD according to tertiles of the model's prediction. AUC denotes area under the receiver operating characteristic curve.

The validation sample consisted of 100 individuals, 62% males, aged 60 ± 13 years, with a 59% prevalence of obstructive CAD. In this group, the AUC was 0.86 (95%CI = 0.79 - 0.93) and Hosmer-Lemeshow's calibration χ^2^ was 10.1 (p = 0.26) (Figure 3A). As the group was divided into tertiles of model's predicted probability (< 30%, 30 - 60%, > 60%), a progressive increase in disease prevalence was observed (24%, 59%, and 94%, respectively, p for trend < 0.001) (Figure 3B).

Compared with the basic model containing only electrocardiogram and troponin (AUC = 0.78), the increment provided by the full model was +0.07 (95%CI of difference between the areas = 0.004 - 0.14, p = 0.039). The integrated discrimination improvement provided by the full model in relation to the first model was 0.09 (p < 0.0015), a result of 0.02 of mean increase of probabilities in the group with events plus 0.07 of mean decrease of probabilities in the group free of events.

### Sensitivity of the final model to electrocardiogram and troponin

The entire sample of 470 patients was utilized to test the model's sensitivity to electrocardiogram and troponin. There was no interaction between the model's prediction and presence (or absence) of electrocardiographic/troponin changes (p = 0.48), meaning that the performance of the model was not modified by these variables. The model's AUC of individuals with normal electrocardiogram and troponin (n = 147, 24% with CAD) was 0.74 (95%CI = 0.65 - 0.83), while individuals with either one abnormal (n = 323, 62% of CAD) had an AUC of 0.77 (95%CI = 0.71 - 0.82).

### Prognostic value for 30-day mortality

**Figure 4:**
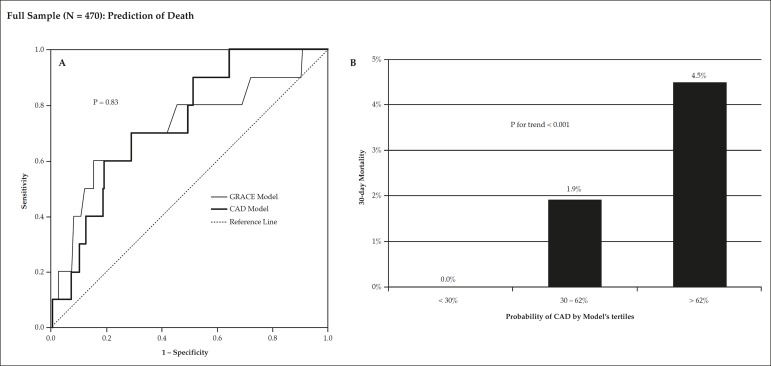
Mortality analysis in the full sample of 470 patients, showing a significant prognostic value of the model, which was originally derived for coronary artery disease (CAD) prediction. Panel A compares the C-index of the model versus GRACE score, indicating similar prediction. Panel B compares the incidence of CAD among tertiles of model's coronary disease prediction. AUC denotes area under the receiver operating characteristic curve.

In the entire sample of 470 patients, 10 patients (2.1%) died within the first 30 days from initial chest pain, eight during hospitalization and two after discharge. The ability of the model to predict death was shown by an AUC of 0.74 (95%CI = 0.61 - 0.87), similar to the GRACE score prognostic value of 0.72 (95%CI = 0.54 - 0.91, p = 0.83) (Figure 4A). There was no death in the first tertile of this entire sample (CAD probability < 30%), three deaths in the second tertile (30 - 62%), and seven deaths in the third tertile (> 62%, p for trend = 0.006) (Figure 4B).

### Acute chest pain score

**Figure 5:**
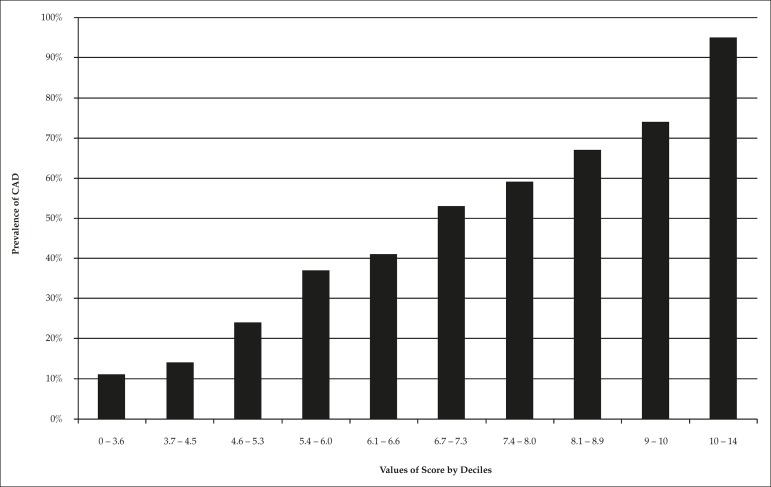
Prevalence of obstructive coronary artery disease (CAD) according to score's deciles.

Points proportional to the regression coefficients were attributed to each positive variable: age (β = 0.025; 0.05 point for each year), relief with nitrates (β = 0.60; 1 point), male gender (β = 1.16; 2 points), ischemic electrocardiogram (β = 1.10; 2 points), positive troponin (β = 1.15; 2 points), and signs of left ventricular failure (β = 1.55; 3 points). The score presented the same AUC as the logistic model. There was a proportional increase in disease prevalence according to score deciles: 11%, 14%, 24%, 37%, 41%, 53%, 59%, 67%, 74%, and 95% (p for linear trend < 0.001) (Figure 5).

## Discussion

The present study developed and validated a probabilistic model to predict obstructive CAD based on data from the initial presentation of acute chest pain. From a total of 35 candidate variables, a final model of six independent predictors was generated, with good discrimination and calibration for assessing the pretest probability of the disease. Most importantly, the accuracy of the model proved to be superior to the traditional model that uses electrocardiogram and troponin.

The indication of diagnostic tests should take into account the pretest probability of the disease. However, in the selected setting of coronary care units, virtually all patients with undefined chest pain undergo testing for detecting obstructive CAD, regardless of pretest probability. Since the test will be negative in a significant proportion of patients,^2^ this approach leads to unnecessarily prolonged hospital stay. Thus, eliminating the need for additional tests in patients with low probability of CAD will improve the efficiency of chest pain protocols. However, validated probabilistic models are not disseminated in this clinical setting, making it hard for the emergency physician to tailor medical decision based on probability. At the most, the probability is evaluated in a binary form, based on whether the electrocardiogram or troponin is altered.

The use of such a probability model improves accuracy and offers a range of continuous probabilities, approximating medical thinking to the best form of dealing with uncertainty. As William Osler once said, "medicine is the science of uncertainty and the art of probability."

Our purpose to predict obstructive CAD should not be confused with previous studies that developed neural or logistic models for predicting the clinical diagnosis of myocardial infarction in patients with chest pain.^8-12^ Such studies created models from clinical data, symptoms characteristics, and sometimes electrocardiogram, which were tested as predictors of a final diagnosis defined by a systematic analysis of the same variables in addition to markers of myocardial necrosis. Therefore, these mathematical models mainly serve as surrogates of medical thinking or, at the most, predictors of a final impression that will be obtained in a few hours of the initial presentation. In contrast, our model was built to predict the result of imaging tests before they are performed. Since noninvasive or invasive imaging tests aim the diagnosis of obstructive CAD, a model of this kind is clearly useful in efficiently selecting patients for these tests, based on the estimation of the pretest probability of the disease. In addition, the knowledge of a pretest probability permits the calculation of the post-test probability after a noninvasive imaging result is obtained.

Other scores focus on the risk of adverse events (HEART score,^13^ TIMI score^14^ or GRACE score^6^). Despite their prognostic value, they are not necessarily good predictors of obstructive CAD^15^ and physicians are uncomfortable to discharge a patient with acute chest pain with no further testing. Thus, we believe that the calculation of the probability of obstructive CAD would encourage physicians to reduce overuse of imaging studies in patients with low probability, diminishing the phenomena of overdiagnosis and overtreatment. For example, patients with a normal electrocardiogram and negative troponin are known to have a good prognosis. In our study, 50% of these patients had a probability of significant CAD below 20%. Based on favorable prognostic and diagnostic probabilities, these patients could be discharged with no further testing. On the other hand, patients with normal electrocardiogram and troponin may have a significant probability of CAD that can be detected by the model. We should point out that future randomized clinical trials should validate the efficiency and safety of this approach.

Physicians normally rely on symptoms characteristics (typical or atypical) and traditional risk factors to estimate the chance of CAD in patients with acute chest pain. For example, a diabetic patient with typical chest pain is usually defined as having a high probability of CAD. However, in our study, no risk factors and chest pain characteristic (except for nitrate relief) independently predicted CAD. This is in agreement with previous studies, which indicate that the type of presentation has little influence on the diagnosis in the acute setting. In a comprehensive systematic review, Swap and Nagurney et al.^16^ showed low likelihood ratios for chest pain characteristics. Seemingly, a recent article by Khan et al.^17^ demonstrated that most pain characteristics are not associated with coronary disease as the cause of the symptom. Therefore, our data reinforce that the approach to rely on risk factors and symptoms to stratify acute chest pain patients has low accuracy. The utilization of a probabilistic model prevents this type of cognitive error.

We purposed three easy forms of utilization of the probabilistic model. First, a score based on points attributed to each positive variable, accompanied by a chart relating summed results and probabilities (Figure 4). Considering the low number of variables, five of them of binary nature, the calculation is easily performed. Second, a logistic score within a spreadsheet with the regression formula, containing age as numeric variable and five "yes" or "no" answers. And, most friendly, an application for smartphones. We believe that by offering different forms of calculations, the clinicians will develop a greater interest in using probabilistic models.

Limitations of the present study should be recognized. The study was performed in a coronary care unit of a specific tertiary hospital, which limits external validity. The population of a chest pain unit is somewhat selected and tends to have a higher prevalence of disease than a general emergency room population. Thus, our model should be further validated for patients with a greater range of clinical presentation. On the other hand, the main purpose of the model is to estimate the pretest probability of hospitalized individuals, which also consist of a large subgroup of real world patients. In this sense, our external validity is not necessarily small; it is just more specific to the tested population.

We should recognize that our sample size is relatively small in comparison with examples of scores delivered from enormous databanks. We have three arguments in favor of our study of 470 patients: first, its novelty as the first successful attempt to develop such a score, which serves at least as a proof of concept that a multivariate model predicts the pretest probability of the disease. Second, in the absence of a multivariate probabilistic model, physicians use clinical judgment based on probabilistic intuition, which has been proved in different settings to be inferior to multivariate models. Thus, considering the remaining alternative of intuition, it might be a good idea to use such a score, not deterministically, but as a tool to avoid common cognitive biases related to intuition. Third, our sample size was based on *a priori* sample size calculation for the logistic regression and for testing the model with ROC curve. According to this calculation, our number of events was enough to provide the minimum power and precision required. Nevertheless, future reports should improve the precision of our estimates.

Finally, among patients who underwent noninvasive tests first, only those with positive results had confirmation by angiography. Nevertheless, predicting a negative noninvasive test (as opposed to no disease at all) is sufficient to prevent the patient from staying unnecessarily to undergo the test.

## Conclusion

The present study developed and validated a novel model to predict obstructive CAD among patients who are admitted with acute chest pain in the coronary care unit. The utilization of such a model should have an impact in preventing overuse of tests, overdiagnosis, and overtreatment while improving the accuracy of pretest assessment of disease probability.
